# Characteristics of the vaginal microbiomes in prepubertal girls with and without vulvovaginitis

**DOI:** 10.1007/s10096-021-04152-2

**Published:** 2021-01-16

**Authors:** Wu Xiaoming, Liu Jing, Pan Yuchen, Liu Huili, Zhang Miao, Shu Jing

**Affiliations:** Department of Pediatric Gynecology, Beijing Children’s Hospital, Capital Medical University, National Center for Children’s Health, Beijing, 100045 China

**Keywords:** Vulvovaginitis, Healthy, Vaginal microbiome, Prepubertal girls

## Abstract

The present study focused on the characteristics of the vaginal microbiomes in prepubertal girls with and without vulvovaginitis. We collected 24 vaginal samples and 16 fecal samples from 10 girls aged 3–9 years with vulvovaginitis and 16 healthy girls of the same age. The samples were divided into three groups: fecal swabs from healthy controls (HF), vaginal swabs from healthy controls (HVS), and vaginal swabs from girls with vulvovaginitis (VVS). Sequencing of the V3–V4 region of the 16S rDNA gene was performed with the NovaSeq PE250 platform to reveal the vaginal microbial community structure in healthy prepubertal girls and vulvovaginitis-associated microbiota. The intestinal microbiomes of healthy children were also analyzed for comparison. This study revealed that the healthy vaginal tract in prepubertal girls was dominated by *Prevotella*, *Porphyromonas*, *Ezakiella*, and *Peptoniphilus* species, with a high diversity of microbiota. The vulvovaginitis-associated microbiota were dominated by *Streptococcus*, *Prevotella*, *Haemophilus*, and *Granulicatella*, with lower diversity than that in healthy girls. Furthermore, the compositions of the vaginal and intestinal microbiomes were completely different. ANOSIM, MRPP, Adonis, and AMOVA were used to analyze the beta diversity, and the results showed that there were significant differences in the microbial communities among the three groups. *Lactobacillus* deficiency and high bacterial diversity were characteristics of the vaginal microbiome in healthy prepubertal girls; this is inconsistent with that in reproductive-age women. The vulvovaginitis-associated vaginal microbiota differed dramatically from normal microbiota, and the main causative agents were not fecal in origin.

## Introduction

Vulvovaginitis, characterized by inflammation of the vulvar and vaginal areas, is the most common gynecological disease in prepubertal girls and frequently causes great anxiety in children and their parents [1]. Anatomic, physiological, and behavioral factors associated with prepubescence create favorable conditions for microorganisms to persist and multiply in the vulva and vagina, causing disruption of vaginal microbiota equilibrium. Disorders involving vaginal flora usually cause infectious clinical syndromes with irritating symptoms, such as vaginal discharge, external genital organ erythema, soreness, itch, irritation, dysuria, and bleeding. Identifying and eradicating the causative pathogen have been the typical clinical diagnosis and treatment practices [2]. Bumbulienė Ž et al. [3] reported that positive microbiological findings were found in 100% of symptomatic girls and in 60% of healthy girls, and fecal bacteria were isolated from 53% of girls with vulvovaginitis and from 25% of healthy girls. *Escherichia coli*, *Enterococcus faecalis*, coagulase-negative *Staphylococcus*, α-hemolytic *Streptococcus*, and group A β-hemolytic *Streptococcus* accounted for 66% of all isolated microbes. In another study, Sikanić-Dugić N et al. reported that among 115 prepubertal girls with vulvovaginitis, causative agents were isolated from vaginal cultures in 38 (33%) cases, of which 21 indicated group A β-hemolytic *Streptococcus*, 5 indicated *Haemophilus influenzae*, 3 indicated *Escherichia coli*, 2 indicated *Enterococcus* spp., and one each indicated *Staphylococcus aureus*, *Proteus mirabilis*, and *Streptococcus pneumoniae* [4]. The main problems in the diagnosis and treatment of vulvovaginitis are that some pathogens cannot be isolated by culture, and it is difficult to determine whether bacteria isolated from the patients’ vaginal secretions are the actual cause of the symptoms or are part of the normal flora. In adult women, modern culture-independent technologies have revealed that the vaginal microbiome is a complex and dynamic ecosystem containing more than 200 bacterial species and that normal vaginal microbiota is mainly dominated by *Lactobacilli* [5]. However, there are few studies on the vaginal microflora in prepubertal girls with and without vulvovaginitis, and additional studies are needed to determine the normal and pathogenic vaginal flora in prepubertal girls [1].

In the present study, we focused on two questions: (i) What are the characteristics of the vaginal microbiome in healthy prepubertal girls? (ii) What are the differences in vaginal microbiome between healthy prepubertal girls and prepubertal girls with vulvovaginitis?

## Materials and methods

### Study setting

The study was performed at the outpatient clinic of the National Center for Children’s Health between February 2020 and April 2020. The study protocol was approved by the Institutional Review Board of the National Center for Children’s Health. Parents of all the study subjects provided written consent for participation in the study and for publication of the study results.

### Inclusion criteria

Ten prepubertal girls (aged 3–9) with vulvovaginitis and 16 healthy girls of the same age were enrolled in the study. The clinical signs of vulvovaginitis were genital redness, vaginal discharge, itch, soreness, rash, irritation, and dysuria. Girls included in the control group had no signs of vulvovaginitis and no history of sexual abuse. All the subjects were prepubescent without secondary sexual characteristics.

### Exclusion criteria

Exclusion criteria were patients with diseases other than vulvovaginitis or a history of sexual abuse.

### Specimen collection

All vaginal specimens were collected using sterile swabs by the same trained pediatric and adolescent gynecologist from the lower third of the vagina. All samples were placed on dry ice immediately after collection and transferred to a − 80 °C freezer within 30 min, where they were stored without any additives until analysis.

### 16S rRNA gene amplification and sequencing

All microbial DNA was extracted from each specimen using the CTAB method, and DNA samples were stored at − 80 °C. Using diluted genomic DNA as a template, specific primers with barcodes and high-efficiency enzymes were used to perform PCR to ensure the efficiency and accuracy of amplification. The PCR primers were 341F (5′-CCTAYGGGRBGCASCAG-3′) and 806R (5′-GGACTACNNGGGTATCTAAT-3′). The library was constructed with the TruSeq® DNA PCR-Free Sample Preparation Kit and quantified by Qubit and Q-PCR. After the library was qualified, NovaSeq6000 was used for sequencing.

### Statistical analyses

According to the barcode sequence and primer sequence for PCR amplification, each sample dataset was differentiated from the offline dataset. After the barcode and primer sequences were removed, the reads of each sample were spliced with Flash, and the splicing sequence was considered the original tag data; the raw tags obtained by splicing were meticulously filtered to obtain high-quality tag data (clean tags). Uparse software was used to cluster all the effective tags from all samples. By default, the sequences were clustered into operational taxonomic units (OTUs) with 97% consistency, and representative OTU sequences were selected. According to the algorithm principle, the OTU sequence with the highest frequency was selected as the representative OTU sequence. The OTU sequences were annotated to obtain taxonomic information. QIIME software was used to calculate the observed OTUs and the Chao1, Shannon, Simpson, and abundance-based coverage estimator (ACE) indices. The dilution curve, rank abundance curve, and species accumulation curve were drawn by R software, and the differences among alpha diversity index groups were analyzed by R software. QIIME software was used to calculate the UniFrac distance and construct the UPGMA sample clustering tree. Principal component analysis (PCA), principal coordinate analysis (PCoA), and nonmetric multidimensional scaling (NMDS) were performed with R software. R software was used to analyze differences in the beta diversity indices among groups. Linear discriminant analysis effect size (LEfSe) software was used for the LEfSe analysis, and the filter value of the LDA score was 4 by default.

## Results

### Bacterial profiles of vaginal and intestinal specimens from healthy girls and vaginal specimens from girls with vulvovaginitis

A total of 26 prepubertal girls aged 3–9, including 10 girls with vulvovaginitis and 16 healthy controls, were enrolled in this study. The 26 subjects contributed 24 vaginal swabs and 16 fecal swabs for analysis. The samples were divided into three groups: fecal swabs from healthy controls (HF), vaginal swabs from healthy controls (HVS), and vaginal swabs from girls with vulvovaginitis (VVS). The 40 swab specimens produced a total number of 2,652,630 reads, averaging 63,158 ± 3142 reads per sample. The mean read length was 416 bp. For the effective tags of all samples, OTU clustering was carried out with 97% consistency, and a total of 1789 OTUs were obtained.

**Fig. 1:**
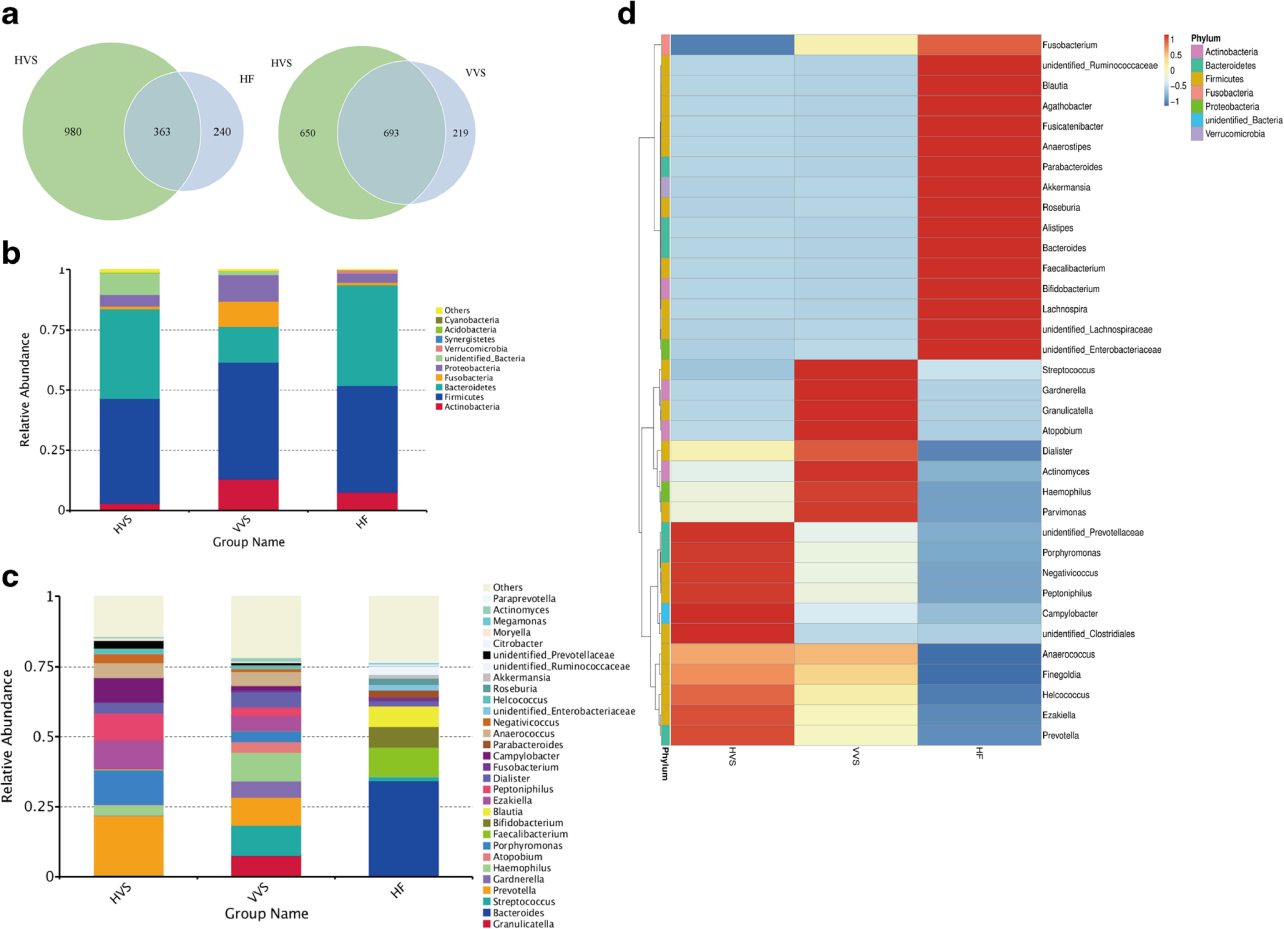
Bacterial profiles of vaginal and intestinal specimens from healthy girls and vaginal specimens from girls with vulvovaginitis. **a** Venn diagrams showing the common and unique OTUs between the HVS and HF groups and between the HVS and VVS groups. **b** Relative abundance of bacteria at the phylum level in the HVS, VVS, and HF groups. **c** Relative abundance of bacteria at the genus level in the HVS, VVS, and HF groups. **d** Relative abundance of each genus in the HVS, VVS, and HF groups

Venn diagrams show the common and unique OTUs between the HVS and HF groups and between the HVS and VVS groups. A total of 1343 and 912 OTUs were detected in the HVS and VVS groups, respectively, compared with 603 OTUs in the HF group. Venn analysis identified 363 common OTUs and 980 and 240 unique OTUs in the HVS and HF groups, respectively, as well as 693 common OTUs and 650 and 219 unique OTUs in the HVS and VVS groups, respectively (Fig. 1a).

The bacterial profile of each group was analyzed, and the results at the phylum and genus levels are shown. The vaginal flora in healthy girls consisted of *Firmicutes*, *Bacteroidetes*, unidentified bacteria, and *Proteobacteria*. The VVS group had a greater abundance of *Actinobacteria*, *Fusobacteria*, and *Proteobacteria* and a lower abundance of *Bacteroidetes* than the HVS group (*P* < 0.05) (Fig. 1b). The gut microbiota in healthy prepubertal girls comprised four major phyla, namely, *Bacteroidetes*, *Firmicutes*, *Actinobacteria*, and *Proteobacteria*. At the genus level, samples from the HVS group were dominated by *Prevotella*, *Porphyromonas*, *Ezakiella*, *Peptoniphilus*, *Campylobacter*, *Anaerococcus*, *Dialister*, and *Haemophilus*. Compared with the HVS group, some of the top 30 bacteria (*Granulicatella*, *Streptococcus*, *Gardnerella*, *Haemophilus*, *Atopobium*) were enriched (*P* < 0.05), while others (*Prevotella*, *Porphyromonas*, *Ezakiella*, *Peptoniphilus*, *Campylobacter*) were depleted in the VVS group (*P* < 0.05) (Fig. 1c, d). The dominant taxa in the HF group were *Bacteroides*, *Faecalibacterium*, *Blautia*, and *Bifidobacterium*, which were not the main vaginal bacteria in children with vulvovaginitis.

These observations suggest that the vaginal microbiome of healthy prepubertal girls is *Lactobacillus* deficient; this result is different from that in reproductive-age women. Vulvovaginitis-associated genera were *Granulicatella*, *Streptococcus*, *Gardnerella*, and so on, which were not of fecal origin.

### Alpha diversity

**Fig. 2:**
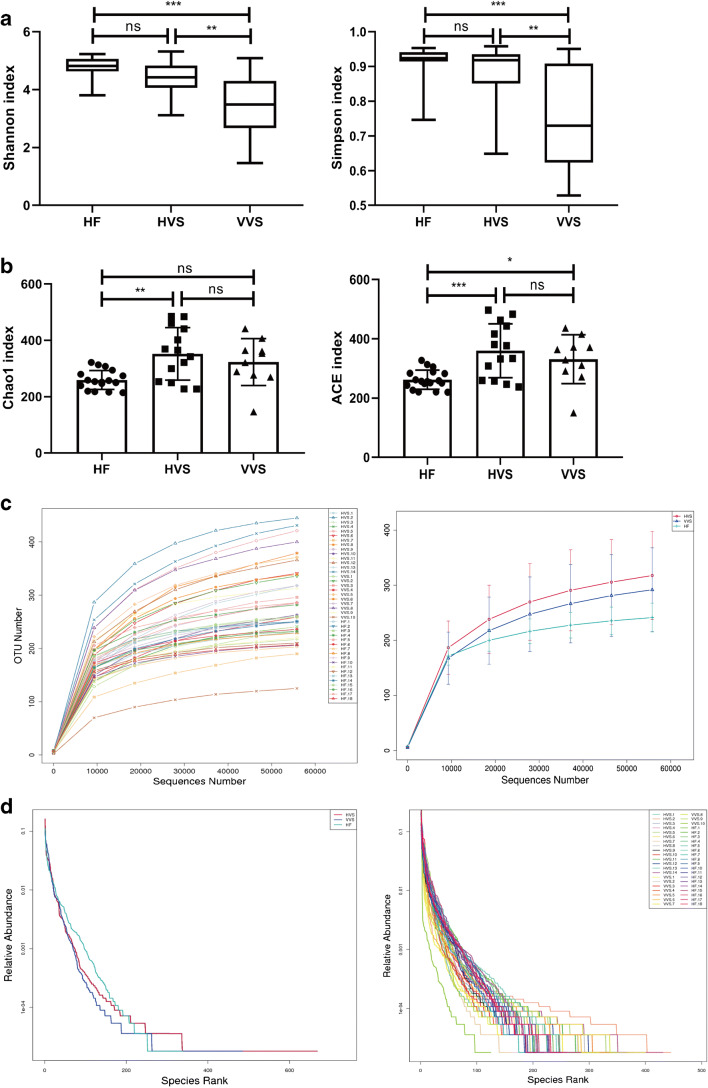
Alpha diversity. **a** Shannon diversity and Simpson diversity at the genus level. **b** Community richness indices—Chao1 index and ACE index. **c** Alpha diversity rarefaction curve. **d** Rank abundance curves

The Simpson index and Shannon index are commonly used to characterize species diversity in a community. They indicate both the richness and evenness of the species present. Neither the Shannon diversity index nor the Simpson diversity index differed between the HF and HVS groups. However, both the Shannon index and Simpson index in the VVS group were significantly lower than those in the HF and HVS groups, indicating that the VVS group had less diversity (Fig. 2a).

The Chao1 richness estimator and ACE were used to estimate the number of OTUs (community richness) in the different samples. The larger the Chao1 and ACE indices are, the higher the richness of the community is. Compared with those in the HF group, the Chao1 index and ACE index in the HVS group increased significantly, but there was no significant difference between these indices in the VVS and HVS groups (Fig. 2b).

A rarefaction curve is often constructed to describe the diversity of the sample species and to indicate whether enough observations have been collected to obtain a good alpha diversity measurement. In Fig. 2c, the upper line represents the HVS group, the middle line represents the VVS group, and the lower line represents the HF group, so we can infer that the flora richness in the HVS, VVS, and HF groups decreased sequentially. Most of the curves did not completely reach the horizontal asymptote, so we may need additional sequencing depth to obtain a good estimate.

The rank abundance curve visually depicts both species richness and species evenness. In Fig. 2d, the red curve representing the HVS group has a long span on the horizontal axis and a shallow gradient, indicating high species richness and evenness. The blue curve representing the VVS group has a short span on the horizontal axis, and the curve drops rapidly and abruptly, indicating that the proportion of dominant bacteria in the sample was high and the diversity was low.

Measures of alpha diversity indicated that the diversity of the vaginal microbiome in healthy prepubertal girls was high; vulvovaginitis-associated dysbiosis is generally characterized by loss of diversity and the presence of pathogens with high levels of abundance.

### Beta diversity

**Fig. 3:**
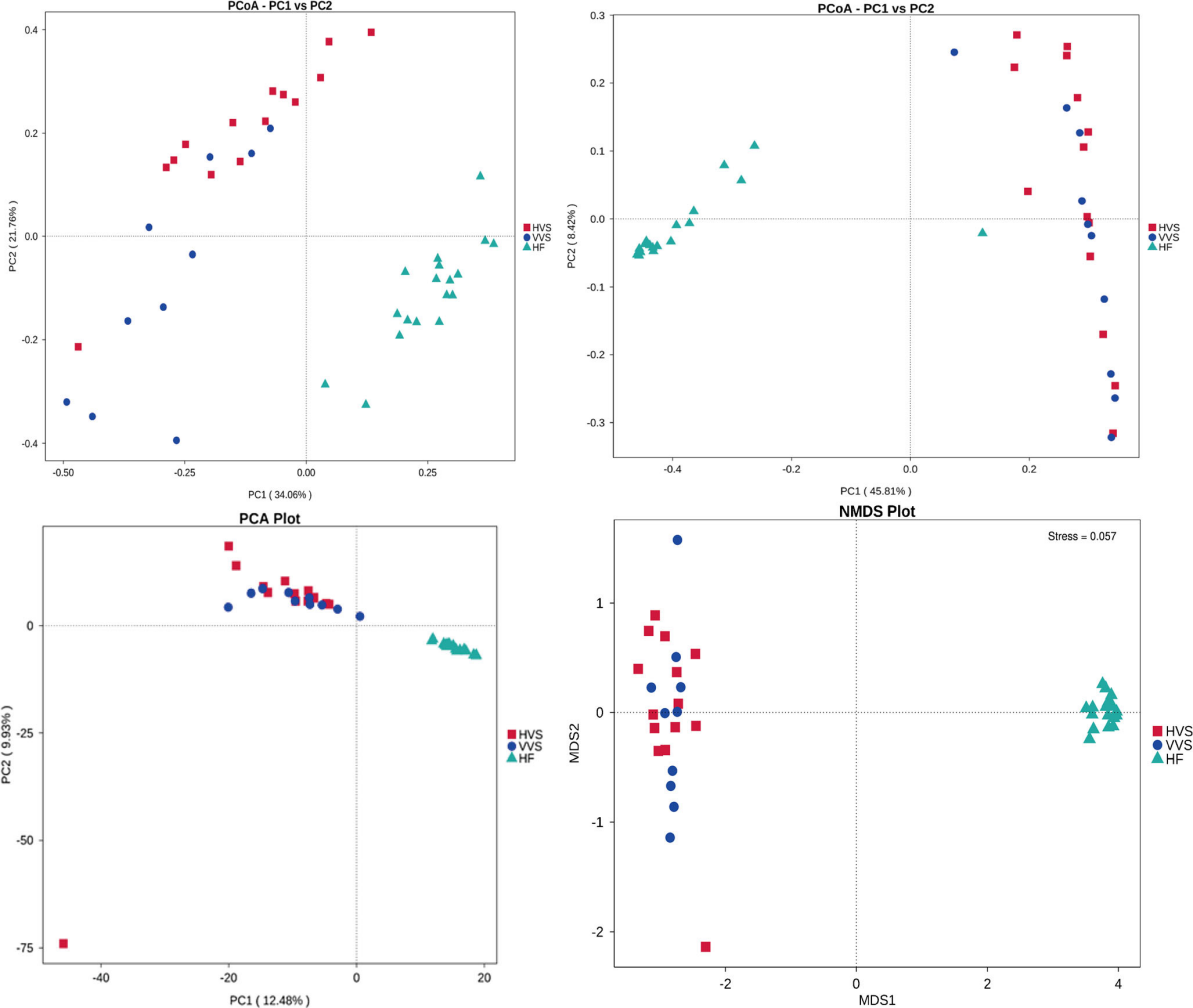
Beta diversity comparisons of microbial communities in the HF, HVS, and VVS groups

Beta diversity measures the variation in species diversity from one environment to another. Figure 3 illustrates the beta diversity measurements of the samples from each participant. The results of PCoA analysis, PCA analysis, and NMDS analysis showed that the intestinal flora in the HF group was significantly different from the vaginal flora in the HVS group and VVS group, but some of the vaginal flora in the HVS group and VVS group could not be completely separated. In addition, through ANOSIM, MRPP, Adonis, and AMOVA analyses, we found that there were significant differences in the bacterial compositions among the HF, HVS, and VVS groups (Tables 1, 2, 3, and 4).

**Table 1:** Analysis of similarities (ANOSIM)

Group	*R* value	*P* value
HF-VVS	0.997	0.001
HVS-VVS	0.3574	0.001
HVS-HF	1	0.001

**Table 2:** Multiple response permutation procedure (MRPP)

Group	*A*	Observed delta	Expected delta	Significance
HF-VVS	0.1649	0.6768	0.8103	0.001
HF-HVS	0.2498	0.5984	0.7976	0.001
HVS-VVS	0.04327	0.6916	0.7229	0.002

**Table 3:** Adonis

Group	*df*	SumsOfSqs	MeanSqs	F.Model	*R*^2^	Pr(>F)
HF-VVS	1 (26)	3.1802 (6.2227)	3.1802 (0.2393)	13.287	0.33821 (0.66179)	0.001
HF-HVS	1 (30)	4.9964 (5.5528)	4.9964 (0.1851)	26.994	0.47363 (0.52637)	0.001
VVS-HVS	1 (22)	0.7879 (5.5457)	0.78794 (0.25208)	3.1258	0.1244 (0.8756)	0.002

**Table 4:** AMOVA

Group	SS	*df*	MS	Fs	*P* value
HF-HVS-VVS	3.20193 (4.21122)	2 (39)	1.60097 (0.10798)	14.8265	< 0.001
HF-HVS	1.93776 (2.44414)	1 (30)	1.93776 (0.0814713)	23.7846	< 0.001
HVS-VVS	0.765319 (3.19523)	1 (22)	0.765319 (0.145238)	5.26942	0.001
HF-VVS	1.93232 (2.78307)	1 (26)	1.93232 (0.107041)	18.0522	< 0.001

### Biomarker taxa for each group

**Fig. 4:**
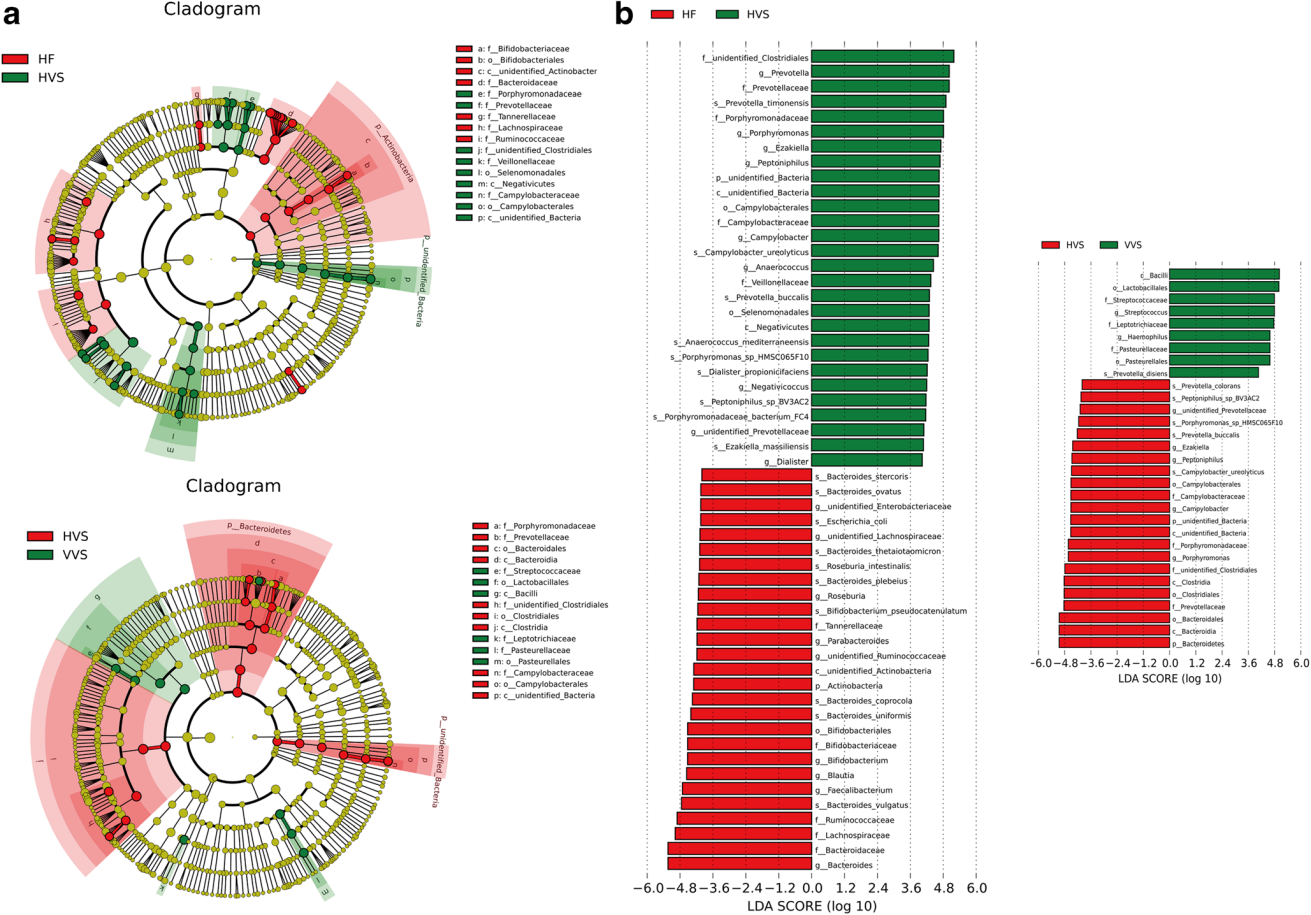
To further define the vaginal microbiota, LEfSe was used to identify taxa in three groups of samples. **a** The cladogram illustrates the taxa with different abundances among the three groups (HVS, VVS, and HF). **b** The distribution histogram of the LDA values shows the species with significant differences in abundance among the different groups

LEfSe was used to predict biomarker taxa in each group (LEfSe *p* values < 0.05 for all taxa listed as enriched) (Fig. 4). The cladogram illustrates the taxa with different abundances among the three groups (HVS, VVS, and HF). At the phylum level, the HVS group was enriched for *Bacteroidetes* (HVS vs. VVS), and the HF group was enriched for *Actinobacteria* (HF vs. HVS). At the genus level, the HVS group was enriched for *Prevotella*, *Porphyromonas*, *Ezakiella*, *Peptoniphilus*, *Campylobacter*, *Anaerococcus*, *Negativicoccus*, and *Dialister* (HVS vs. HF). In contrast, the dominant taxa in the VVS group samples were more likely to be pathogenic, e.g., *Streptococcus* and *Haemophilus* (VVS vs. HVS).

## Discussion

Although the clinical symptoms and signs associated with vulvovaginitis in prepubertal girls are relatively easily measured, it is difficult to achieve consistent detection to verify the cause of vulvovaginitis [7]. It has been implicated that vulvovaginitis, a common problem in pediatric and adolescent gynecology, is most likely caused by disruption of vaginal microbiota equilibrium. Multiple studies to determine the vaginal flora of healthy prepubertal girls and vaginal flora dysbiosis in girls with vulvovaginitis have been performed using traditional methods of bacterial culture. However, the vaginal flora composition of prepubertal girls has not been well defined [8]. The major purpose of this study was to investigate the characteristics of the vaginal microbiome in healthy prepubertal girls, as well as vulvovaginitis-associated vaginal microbiota in prepubertal girls with 16S ribosomal RNA amplicon sequencing.

The first step was identifying the compositions of bacterial communities. According to the literature, the vaginal microbiota in asymptomatic reproductive-age women can be categorized into five major bacterial community groups with distinct diversity and species compositions. Most vaginal communities are dominated by the *Lactobacillus* genus, which is able to produce lactic acid that consequently reduces vaginal pH and thus facilitates an acidic environment in the vagina [9]. In our study, we found that at the genus level, the vaginal flora in healthy girls was dominated primarily by *Prevotella*, *Porphyromonas*, *Ezakiella*, *Peptoniphilus*, and *Fusobacterium*. *Prevotella*, not *Lactobacillus*, was the predominant vaginal species in healthy prepubertal girls, although there is evidence that several species of *Prevotella* found in the vagina are associated with bacterial vaginosis (BV) [10]. Compared with the HVS group, the relative abundance of *Granulicatella*, *Streptococcus*, and *Haemophilus*, which have been found to be closely related to vaginal infection, was enriched in the VVS group [11, 12]. It has been reported that fecal flora is a significant cause of vulvovaginitis in prepubertal girls. To demonstrate the cause of the infection, fecal microbiome data was used to make a correlation. The gut microbiota in an infant appears unstructured, and it starts resembling adult microbiota by the age of 3 years. Our data showed that the composition of gut microbiota in healthy prepubertal girls was consistent with that in healthy adults and mainly contained *Bacteroidetes*, *Firmicutes*, *Actinobacteria*, and *Proteobacteria* [6].

Alpha diversity, representing the microbial diversity within each sample, was analyzed based on the OTU richness, the Shannon index, and the inverse Simpson index. In women of reproductive age, vaginal health includes vaginal microbiota with low diversity that is predominated by *Lactobacillus*. BV and high-risk sexual behavior are associated with increased diversity in the vaginal microbiome and a lack of *Lactobacillus* [13]. Unlike that in women of childbearing age, the vaginal microbiota in healthy prepubertal girls was highly diverse, similar to gut microbiota, while the microbial diversity in girls with vulvovaginitis was depleted significantly according to our results. To compare the bacterial community structures, we clustered all samples using PCoA, PCA, and hierarchical clustering (Bray-Curtis dissimilarities). At the OTU level, all samples strongly clustered according to vaginal or intestinal origins and rendered microbiota significantly different among the HF group, HVS group, and VVS group.

## Conclusions

In this study, we found that the vaginal microbiome of healthy prepubertal girls was dominated by *Prevotella*, *Porphyromonas*, *Ezakiella*, and *Peptoniphilus*, with a high microbial diversity, which is inconsistent with the normal vaginal community structure of the majority of reproductive-age women. The vaginal microbiota associated with vulvovaginitis was less diverse, with an increased abundance of *Granulicatella*, *Streptococcus*, and *Haemophilus*. Furthermore, bacteria of fecal origin may not be the pathogen responsible for vulvovaginitis in prepubertal girls.

## Data Availability

The data used to support the findings of this study are available from the corresponding author upon request.
